# Robust Visual Tracking with Reliable Object Information and Kalman Filter

**DOI:** 10.3390/s21030889

**Published:** 2021-01-28

**Authors:** Hang Chen, Weiguo Zhang, Danghui Yan

**Affiliations:** Automation College, Northwestern Polytechnical University, Xi’an 710072, China; zhangwg@nwpu.edu.cn (W.Z.); yandh@mail.nwpu.edu.cn (D.Y.)

**Keywords:** visual object tracking, correlation filter, reliable information, Kalman filter

## Abstract

Object information significantly affects the performance of visual tracking. However, it is difficult to obtain accurate target foreground information because of the existence of challenging scenarios, such as occlusion, background clutter, drastic change of appearance, and so forth. Traditional correlation filter methods roughly use linear interpolation to update the model, which may lead to the introduction of noise and the loss of reliable target information, resulting in the degradation of tracking performance. In this paper, we propose a novel robust visual tracking framework with reliable object information and Kalman filter (KF). Firstly, we analyze the reliability of the tracking process, calculate the confidence of the target information at the current estimated location, and determine whether it is necessary to carry out the online training and update step. Secondly, we also model the target motion between frames with a KF module, and use it to supplement the correlation filter estimation. Finally, in order to keep the most reliable target information of the first frame in the whole tracking process, we propose a new online training method, which can improve the robustness of the tracker. Extensive experiments on several benchmarks demonstrate the effectiveness and robustness of our proposed method, and our method achieves a comparable or better performance compared with several other state-of-the-art trackers.

## 1. Introduction

Visual object tracking is one of fundamental problems in the field of computer vision. This task aims to estimate the target location in all frames after the initial frame target is given. It has been widely used in many aspects of real life, including video surveillance [1], human-computer interaction [2], robots [3] and automatic drive [4]. In recent years, this field has attracted a large number of researchers and a lot of excellent works [5,6] have also emerged. Although great progress has been made in visual tracking recently, visual object tracking is still an open problem in the field of computer vision because of the challenging scenarios (e.g., deformation, illumination variation, occlusion, background clutter, etc.) in tracking process.

Recently, correlation filter (CF) [7,8,9,10] methods have attracted a lot of attention, which have the advantages of accurate tracking precision and high tracking frame rate. CF methods use cyclic shift to approximate dense sampling, which greatly increases the number of training samples, solves the problem of training samples shortage. Additionally, according to the convolution theorem, convolution operation of correlation filter is converted to frequency domain for calculation, which greatly reduces the computational complexity and enhances the real-time performance. Although the CF tracking methods have these advantages, there are still some drawbacks. Most CF methods adopt simple linear interpolation to update the model, which will lead to two problems. First, the reliability of tracking results are not analyzed. When facing challenging scenarios (e.g., occlusion, background clutter, aspect ratio change, etc.), the noise information is gradually added to the filter when online training and updating in tracking process and the model will be distorted. Second, the first frame information in the whole tracking process is the most reliable. However, with the updating process, the first frame information in the model gradually decreases, which reduces the robustness of the model. In addition, most CF methods do not consider the relationship between frames.

**Figure 1:**
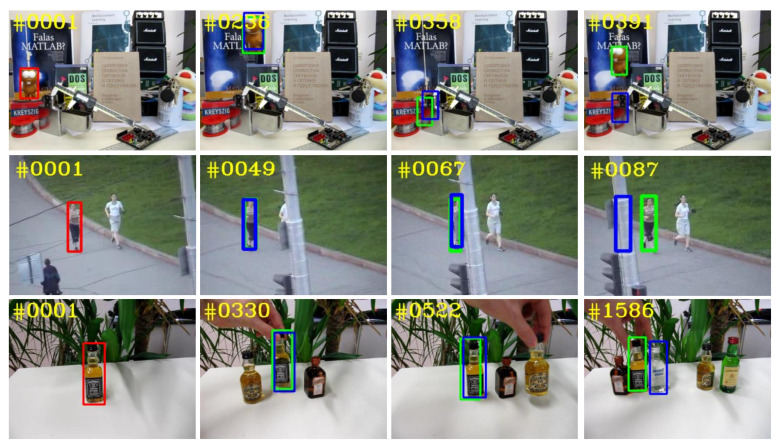
Tracking results in challenging scenarios. The first column is the initial frame, in which the red box object specifies the target to be tracked. The following columns are the tracking results in complex scenes, and the blue box represents the basic tracker, and the green box represents ours.

In order to address these problems, we propose a robust visual tracking framework based on reliable object information and Kalman filter. The method in this paper mainly includes three modules: tracking results reliability analysis (TRRA) module, Kalman filter (KF) module and reliable online training (ROT) module. As for the noise interference problems in online training, the TRRA module will analyze the tracking results and select the most reliable object information for model training to reduce the impact of noise. For the problem of the decline of the object information in the first frame of the model, we propose a new model training method, which uses the first frame and the current frame jointly to train the model to improve the stability of the model. Finally, we use the KF module to model the object motion information, so as to supplement the CF tracking and improve the tracking robustness. Figure 1 shows that our tracker can handle complex tracking scenarios and has better tracking performance than the basic tracker CFNet [11].

The main contributions of this method can be summarized as follows:

- We propose a new reliable online training method, which can preserve the useful first frame target information.
- We develop a Kalman filter to describe the object’s motion information, then use the trajectory information to guide the tracking process.
- We propose a reliability analysis method for tracking process. This ensures the validity of the target information in the model training process.
- Extensive experiments are conducted on several benchmark datasets. The results show the effectiveness and robustness of the proposed method. In addition, our method achieves a competitive tracking performance compared with other state-of-the-art trackers.

This paper is organized into 5 sections. Some related works are summarized in Section 2 and the proposed method in this paper is described in Section 3. The experimental results are provided in Section 4. Section 5 is the conclusion of this paper.

## 2. Related Work

### 2.1. Correlation Filter Methods

The method based on correlation filter was pioneered by Bolme et al. in MOSSE [12]. MOSSE is a linear discriminant classifier based on single-channel pixel feature, and achieves the frame rate over 600 FPS. Many improved CF methods have also been proposed subsequently. KCF [7,13] introduces the kernel technique into the CF methods to improve the discriminative ability of the classifier. Multi-channel features also greatly improve the tracking performance of CF methods, such as KCF uses Histogram of Oriented Gradient (HOG) features, SAMF [14] uses HOG and CN features. DSST [15] uses a one-dimensional correlation filter and multi-scale template to accurately estimate the target scale, which solves the problem of target scale variations and wins the championship on VOT2014. In order to solve the problem of training correlation filters limited in small search area, SRDCF [8] adds a space penalty term to the optimization objective function, which enables the filter to track the target in a larger searching area and reduces the boundary effect of correlation operation. With the introduction of deep convolution features by DeepSRDCF [16], the performance of SRDCF tracker has been further improved. C-COT [10] learns discriminative convolution operators and obtains confidence map of the target all in continuous space domain, to improve the richness of model and the localization accuracy. In consideration of the great influence of background information on tracking performance, Mueller et al. [17] propose a tracking framework to explicitly learn the background information around the target on CF trackers. This framework can be widely used in CF trackers to improve the tracking performance. Bibi et al. [18] proposed an adaptive target response framework, which can adaptively change the target response frame by frame, making the tracker insensitive to error locations. Xia et al. [19] build a tracker with fused deep features and correlation filters to solve challenge situations.

### 2.2. Deep Learning Methods

Recently, deep learning framework have been used in the field of visual tracking. Since deep learning has the characteristics of large training data sets and computational requirements, the trackers based on deep learning can be divided into two categories. One is to use convolutional neural network (CNN) pretrained on other data sets as feature extractor, and then combine with traditional methods to achieve object tracking. As mentioned in the previous subsection, DeepSRDCF [16], C-COT [10] and ECO [20] combine the deep features extracted by pretrained CNN with CF, and achieve the state-of-the-art tracking performance. The other is to fully adopt deep learning structure, and then train the tracker end-to-end on large data sets. MDNet [21] proposes a multi-domain learning model based on CNN, which can separate the independent information of multiple targets from the target. GOTURN [22] uses the image pairs of the previous frame target and the current frame search area as input, and then directly regresses the position of the target in the search area through the deep network. It can achieve the tracking frame rate of 100FPS. SINT [23] and SiamFC [24] formulate the visual tracking as a similarity learning problem. By training a similarity matching network on the detection dataset, the target in the first frame is compared with the candidate regions of the subsequent frame to realize the target estimating. There is no model updating in the tracking process, so they achieve both high frame rate and high tracking accuracy. The backbone used in SiamFC is relatively shallow, SiamDW [25] and SiamRPN++ [26] explore deeper networks to improve tracking performance. CFNet [11] takes CF as a differentiable layer of deep neural network to realize the end-to-end training of the network. SANT [27] presents structure-attention networks to learn robust structure information of targets. HKSiamFC [28] adopts Histogram model to explore target’s prior color information, and makes SiamFC more robust in some complex environments.

### 2.3. Temporal Stability

Making full use of temporal information is very important for the robustness of visual object tracking. Many methods using temporal information are proposed to improve tracking performance. One kind of tracker simply uses temporal information, such as CF [7,8,13] and some deep trackers [11,23,24], by focusing on the region near the target in the previous frame and suppressing other remote regions. The other kind of tracker is to encode temporal information directly by Recurrent Neural Network (RNN) [29] or Long Short-Term Memory (LSTM) [30,31]. In this paper, we use KF to model the object motion, and then use the temporal information of video sequence to supplement the tracking process.

## 3. Proposed Method

**Figure 2:**
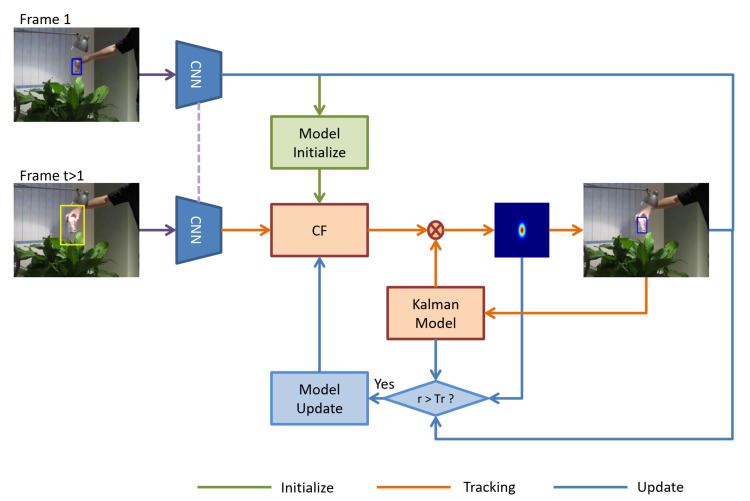
Pipeline of our proposed method. Firstly, the correlation filter is initialized with target information in the first frame. Secondly, the correlation filter (CF) and Kalman filter are used to estimate the target location in the subsequent frames, and then the reliability of the estimation is analyzed by reliability analysis module. Once the localization is reliable, the model is trained jointly by using the target information in the initial frame and the current reliable information. Finally, CF is updated. Besides, *r* is the reliability of the tracking result; $T_{r}$ is the threshold to determine whether the tracking is reliable. In addition, we use CNN to extract image features.

In this section, we will elaborate on the method proposed in this paper, which mainly includes three components: reliability analysis, Kalman filter, and reliable online training method. Finally, we also introduce the filter update and tracking details. The overview of our proposed method is shown in Figure 2.

### 3.1. Reliability Analysis

**Figure 3:**
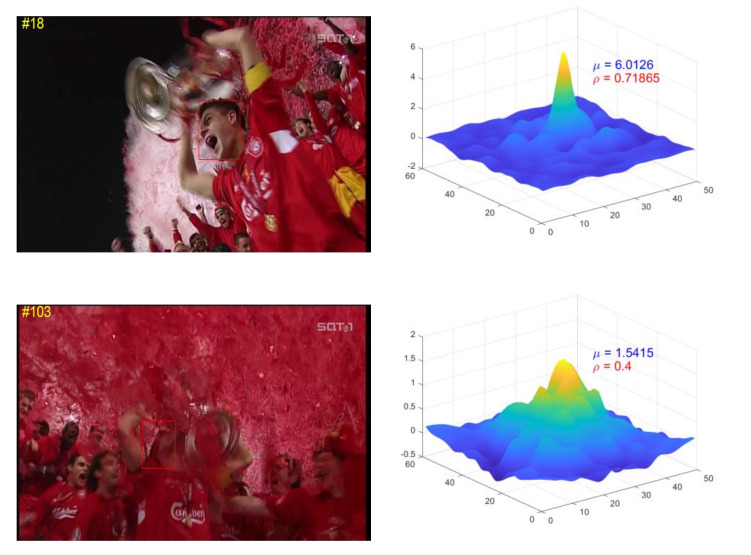
The imagesin first column are frames of sequence Soccer, and corresponding precision reliability and stability reliability are placed in the second column. Obviously, the reliability of target image in first row is higher than that in second row.

The response map represents the tracking result on the current frame. So we can calculate the reliability of the tracking process by analyzing the response map. Response reliability can be analyzed through two aspects: precision and stability as shown in Figure 3. Intuitively, a larger maximum response corresponds to a higher accurate location. The precision corresponds to the magnitude of response of the correlation filter. So the precision reliability is expressed as

(1) $$\mu_{l}=\max\left(S_{l}\right),$$

where $S_{l}$ represents the response map of frame *l*.

Stability reliability corresponds to the quality of filtering response. Peak Sidelobe Ratio (PSR) is mentioned in the MOSSE tracker as a criterion to measure detection process. PSR indicates the quality of filtering response and whether tracking drift occurs. It calculate the ratio of sub-peak to main peak to estimate the reliability of tracking process.

(2) $$PSR=\frac{r_{sub}}{r_{main}},$$

where $r_{sub},r_{main}$ represent sub-peak and main peak respectively.

However, this method has one problem, it can not deal with the problem of similar object interference. For example, when a new similar object appears, there may be a higher sub-peak around the main peak, resulting in a larger PSR value, but this does not indicate that the tracking fails, and the tracking result is still reliable. So we improve the stability reliability calculation as follows:

(3) $$\rho_{l}=1-\min\left(\frac{r_{mean}}{r_{max}},0.6\right),$$

where $r_{max}$ and $r_{mean}$ are the main peak and the mean value of response map respectively. Threshold 0.6 is also used to mitigate the penalty when similar objects appear in the search image.

From Equations (1) and (3), we can calculate the final tracking process reliability

(4) $$r_{l}=\min\left(\frac{\mu_{l}}{\mu_{max}}\cdot \rho_{l},1\right),$$

where $\mu_{max}$ is the maximum value of all response maps that have been tracked. When the target response reliability meets the condition of $r_{l}>T_{r}$, it indicates that the tracking process is reliable. $T_{r}$ is a reliability threshold, which is set to 0.6 in this paper.

### 3.2. Trajectory Modeling and Kalman Filter

Most of the traditional tracking methods [7,8,11,15,24] only focus on the target detection in the current frame in the tracking process, and rarely model the temporal information of the target between frames. Visual tracking is based on video image sequence, so temporal information is very important for robust tracking, especially in challenge tracking scenes such as occlusion and the existence of similar distractors. In this paper, we use KF to model the motion of the object and get the trajectory information.

Kalman filtering (KF) is an algorithm that uses the state equation of linear system to estimate the system state through the input and output observation data of the system. Given the system parameters, initial values and measurement sequences, the KF can estimate the system state sequences iteratively. For the tracking tasks, because there is no control variable, we can first ignore the input, and the process noise and observation noise can be set as white noise. The label given in the initial frame is a bounding box (x,y,w,h). In the motion model, we only consider the position information, so we can formulate the system state as a 4-dimensional vector $\left(x_{c},y_{c},v_{x},v_{y}\right)$, where $x_{c},y_{c}$ represent the object center, $v_{x},v_{y}$ is the velocity in both directions. In this paper, we approximate the translation between frames as a constant velocity model.

(5) $$\left\{\begin{array}{c} X_{k}=FX_{k-1}\\ Z_{k}=HX_{k}+V_{k} \end{array}\right.$$

where *F* is the state transition matrix, *H* is measurement matrix, $V_{k}$ is measurement noise, and $Z_{k}$ is the measurement.

The process of state estimation can be divided into two steps: prediction step and update step. We can use the following formulas to perform the prediction step:

(6) $${\hat{X}}_{k,k-1}=F{\hat{X}}_{k-1},$$

(7) $$P_{k,k-1}=FP_{k-1}F^{T},$$

where ${\hat{X}}_{k-1}$ is the optimal estimation of the previous state, $P_{k-1}$ is the error covariance matrix of the previous optimal state estimation.

The formula for calculating the Kalman gain is as follows:

(8) $$K_{k}=P_{k,k-1}H^{T}\left(HP_{k,k-1}H^{T}+R\right),$$

where *R* is the measurement error covariance matrix.

The measurement in this paper can be set as the output of CF. At last, the predicted values can then be updated:

(9) $${\hat{X}}_{k}={\hat{X}}_{k,k-1}+K_{k}(Z_{k}-H{\hat{X}}_{k,k-1}),$$

(10) $$P_{k}=\left(I-K_{k}H\right)P_{k,k-1},$$

where ${\hat{X}}_{k}$ is the posterior estimation of current state, *I* is the identity matrix, and $P_{k}$ is the error covariance matrix of the current state estimation. Thus, we get the optimal state estimation of the current step through the motion model. The optimal estimation can be regarded as a refined update of CF tracking results in visual tracking task. It can be a powerful supplement to CF tracking method.

### 3.3. Reliable Online Training

The first frame contains the only absolutely reliable information of the target. Maintaining the first frame information in the tracker is very important for robust tracking. In this paper, we combine the first frame target information with the current target information to train a reliable correlation filter.

Firstly, we review the traditional CF tracking methods. The principle of CF method is extremely simple while tracking at very high frame rate and maintaining high tracking performance. The core advantages of this method lie in two points: (1) A Large number of approximate samples are obtained by intensive sampling of the original signal through cyclic shift. (2) In the process of training and detection, correlation operations are converted into frequency domain, to simplify the calculation greatly. The CF methods reformulate the tracking process as a ridge regression problem, train the filter through the samples and labels, and then use the filter to locate the target in search patch and update the filter on newly located object. The objective function of ridge regression can be expressed as follows:

(11) $$\min_{w}{∥Xw-y∥}^{2}+\lambda_{1}{∥w∥}^{2},$$

where sample matrix *X* contains the data vector *x* and all its cyclic shift versions as row vector, *w* is the correlation filter to be learned, *y* is the Gaussian shape regression response corresponding to all samples, $\lambda_{1}$ is a regularization parameter to prevent over-fitting of the model.

Traditional CF trackers use linear interpolation to update the filter, which makes the reliable initial target information decrease exponentially. These methods are effective for tracking under simple situation. For challenging scenarios, noise information will distort the learned filter, which decline the tracking performance or even lead to tracking drift. We use reliable object information in initial frame and current object information to enhance target foreground information and reduce the impact of noise.

Suppose that each target image $X_{l}$ has M-dimensional features $X_{l}^{i},i=1,\ldots ,M$, the corresponding filter for each feature channel is $w_{i},i=1,\ldots ,M$. We reformulate Formula (11) to

(12) $$\min_{w}\sum_{l=1}^{2}\beta_{l}{∥\sum_{i=1}^{M}X_{l}^{i}w_{i}-y∥}^{2}+\lambda_{1}\sum_{i=1}^{M}{∥w_{i}∥}^{2},$$

where $\beta_{l},l=1,2$ are the weights for the templates.

The summation formula can also be written in vector form

(13) $$\min_{w}{∥\bar{X}\bar{w}-\bar{y}∥}^{2}+\lambda_{1}{∥\bar{w}∥}^{2},$$

$$\begin{array}{c} \bar{X}=\left[\begin{array}{ccc} \sqrt{\beta_{1}}X_{1}^{1} & \cdots & \sqrt{\beta_{1}}X_{1}^{M}\\ \sqrt{\beta_{2}}X_{2}^{1} & \cdots & \sqrt{\beta_{2}}X_{2}^{M} \end{array}\right],\bar{w}=\left[\begin{array}{c} w_{1}\\ \vdots \\ w_{M} \end{array}\right],\bar{y}=\left[\begin{array}{c} \sqrt{\beta_{1}}y\\ \sqrt{\beta_{2}}y \end{array}\right]. \end{array}$$

Formula (13) can be solved by setting the gradient of the objective function to zero

(14) $$\bar{w}={\left({\bar{X}}^{T}\bar{X}+\lambda_{1}I\right)}^{-1}{\bar{X}}^{T}\bar{y}.$$

According to the properties of cyclic matrix, fast calculation is carried out in frequency domain. The solution in frequency domain is as follows

(15) $$\hat{\bar{w}}={\left[\begin{array}{ccc} D_{11}+\lambda_{1} & \cdots & D_{1M}\\ \vdots & \ddots & \vdots \\ D_{M1} & \cdots & D_{MM}+\lambda_{1} \end{array}\right]}^{-1}\left[\begin{array}{c} \sum_{l=1}^{2}\beta_{l}{\left(X_{l}^{1}\right)}^{*}⊙\hat{y}\\ \vdots \\ \sum_{l=1}^{2}\beta_{l}{\left(X_{l}^{M}\right)}^{*}⊙\hat{y} \end{array}\right],$$

$$D_{ji}=\sum_{l=1}^{2}\beta_{l}diag\left({\left({\hat{X}}_{l}^{j}\right)}^{*}⊙{\hat{X}}_{l}^{i}\right),j,i=1,\ldots ,M,$$

where ⊙ represents elment-wise multiplication, * denotes complex conjugate. The variable with hat represents its corresponding Fourier transform.

For multi-channel, the primal domain detection needs to use the corresponding filter to detect in each channel of search image *Z*, and finally all the channel detection results are integrated

(16) $$\hat{f}\left(Z\right)=\sum_{i=1}^{M}{\hat{z}}_{i}⊙{\hat{w}}_{i}.$$

The solution in dual space is

(17) $$\bar{\alpha }={\left(\bar{X}{\bar{X}}^{T}+\lambda_{1}I\right)}^{-1}\hat{y}.$$

Then, according to the properties of cyclic matrix, it is converted to frequency domain for calculation

(18) $$\hat{\bar{\alpha }}={\left[\begin{array}{cc} D_{11}+\lambda_{1} & D_{12}\\ D_{21} & D_{22}+\lambda_{1} \end{array}\right]}^{-1}\left[\begin{array}{c} \sqrt{\beta_{1}}\hat{y}\\ \sqrt{\beta_{2}}\hat{y} \end{array}\right],$$

where

$$D_{jl}=\sum_{i=1}^{M}\sqrt{\beta_{j}\beta_{l}}diag\left({\hat{X}}_{j}^{i}⊙{\left({\hat{X}}_{l}^{i}\right)}^{*}\right),j,l=1,2.$$

At last, the detection formula is

(19) $$\hat{f}\left(Z\right)=\sum_{l=1}^{2}\sum_{i=1}^{M}{\hat{z}}^{i}⊙{\left({\hat{X}}_{l}^{i}\right)}^{*}⊙\hat{\alpha_{l}}.$$

### 3.4. Filter Update

Most traditional CF trackers adopt strict frame-by-frame update strategy. However, the target information between adjacent frames changes little and has much redundant information, which not only slows down the tracking speed, but also makes the tracking performance degraded when facing complex tracking environment. Many researchers also proposed improved method to update every N frames, but it still exist the problem of inaccurate object information. In our method, we adopt the strategy of sparse updating and reliability analysis of target information. Therefore, we can get more robust and accurate updated filters. To obtain a better performance and avoid drastic change of model, we use a moving average method to update correlation filter.

(20) $${\hat{w}}_{i}^{t}=\left(1-\delta \right){\hat{w}}_{i}^{t-1}+\delta {\hat{w}}_{i}^{new},$$

(21) $${\hat{\alpha }}_{l}^{t}=\left(1-\eta \right){\hat{\alpha }}_{l}^{t-1}+\eta {\hat{\alpha }}_{l}^{new},$$

where δ,η are the corresponding learning rates.

### 3.5. Tracking Details

**Figure 4:**
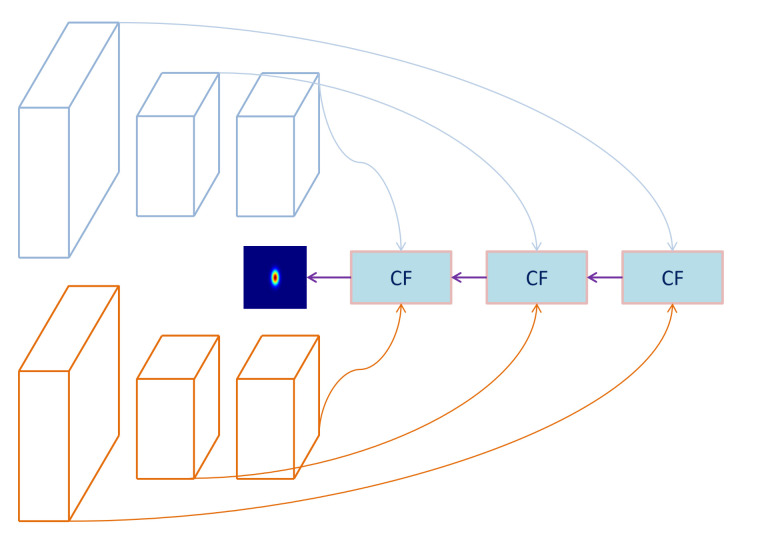
Details of the fusion process of response maps. The blue block represents the correlation filter. First, the three stage features are fed into three correlation filters to obtain three response maps, and then all the results are fused to compute the final location estimation.

Deep features are extracted by VGG-Net-19 network [32] which removes all the full-connection layers. The network is pre-trained on the ImageNet [33] ILSVRC dataset to perform classification tasks, and the deep features extracted by VGG have also been used in many other fields [11,34]. Instead of just using the output of the last layer of the network, we use the output of 3-4, 4-4 and 5-4 layers. This is because the high-level features tend to be semantic, with high stability but low resolution, which is conducive to improve the robustness of the tracking process. The low-level features are more texture oriented, with low stability but high resolution, which is conducive to improve the accuracy of localization process. As shown in Figure 4, we calculate the response maps on the features of three layers. Finally, we fuse the three response maps to get the final result,

(22) $$S_{f}=\sum_{l=3}^{5}w_{l}S_{l},$$

where $S_{f}$ is the fused result, $S_{l},l=3,4,5$ represent response maps of different layer features. $w_{l}$ is the weight for fusing.

In order to improve the accuracy and stability of the tracking process and make the filter more suitable for future tracking targets, we use the reliability values of current frame to calculate the weight

(23) $$\beta_{1}=\gamma_{l},\;\beta_{2}=1-\beta_{1}.$$

The detailed tracking method is shown in Algorithm 1.
**Algorithm 1** Robust Visual Tracking with Reliable Object Information and Kalman Filter**Input:** Initial target position $p_{0}$ **Output:** Estimated target position $p_{t}$ and updated correlation filters
 1:Initialize the filters according to $p_{0}$, and save object features $X_{0}$ 2:**repeat** 3: According to the $p_{t-1}$ and correlation filters, calculate the ${\bar{p}}_{t}$ in frame *t*; 4: Taking the computed ${\bar{p}}_{t}$ as observation, estimate the target position ${\hat{p}}_{t}$ by Kalman filter; 5: Fuse the results of two modules, and obtain $p_{t}$; 6: According to the fusion confidence map, analyse the reliability of the tracking process; 7: **if** reliability>Threshold **then** 8:  Send $X_{0},X_{t}$ into the online training module, and update the filters; 9: **else**
 10:  Continue; 11: **end if**
 12:**until** The last frame of the sequences


## 4. Experiments

In this section, we firstly demonstrate the effectiveness of this method with ablation experiments. Then, we compare our proposed method with state-of-the-art trackers on dataset OTB-2013 [35], OTB-2015 [36], VOT2016 [37], and VOT2018 [38].

### 4.1. Evaluation Criteria and Parameter Setting

On OTB dataset, we use OPE criterion [5,37] to evaluate all trackers, which including two metrics: precision and success rate. Precision is the Euclidean distance between the center position of estimated result box and ground truth bounding box. Twenty pixel distance threshold is usually used to compare the performance of each tracker. Success rate is a measure of the overlapping area of two boxes

(24) $$IOU=\frac{area(B_{T}\cap B_{G})}{area(B_{T}\cup B_{G})},$$

where $B_{T}$, $B_{G}$ are the estimation and ground truth respectively, ∩,∪ denote the intersection area and union of two boxes. When the overlap area exceeds a certain threshold, such as IOU≥0.5, we assume that the tracking in this frame is successful. The success rate can be obtained by dividing the number of frames successfully tracked by the total number of frames. Area Under Curve (AUC) value is usually used to ranking the trackers in success plot.

In the VOT protocol, the trackers need to be reinitialized when tracking fails. Trackers performance is measured by accuracy and robustness, which correspond to the bounding box average overlap during successful tracking and failure rate, respectively. Expected Average Overlap (EAO) is used to evaluate the overall tracker performance. Please refer to VOT2016 [37] for details.

We have implemented the proposed method in MATLAB, in which the implementation of convolution neural network is based on MatConvNet toolbox [39]. All trackers run on the same computer equipped with Intel Core i7-8700 CPU, 16GB RAM and a NVIDIA GTX 1080 GPU.

### 4.2. Ablation Experiments

In this section, we conduct ablation experiments on OTB dataset, and analyze the effectiveness of each module proposed in this paper. We use DCF as the baseline tracker, but the difference is that we use convolution network to extract features. In order to test the performance of different components, we build three different trackers using baseline tracker and each component: (1) Baseline+RA is constructed by baseline and reliability analysis module, (2) Baseline+KF is constructed by baseline and Kalman Filter, (3) Baseline+OT indicates that the updated filter is trained by target information of the first frame and the current frame.

**Figure 5:**
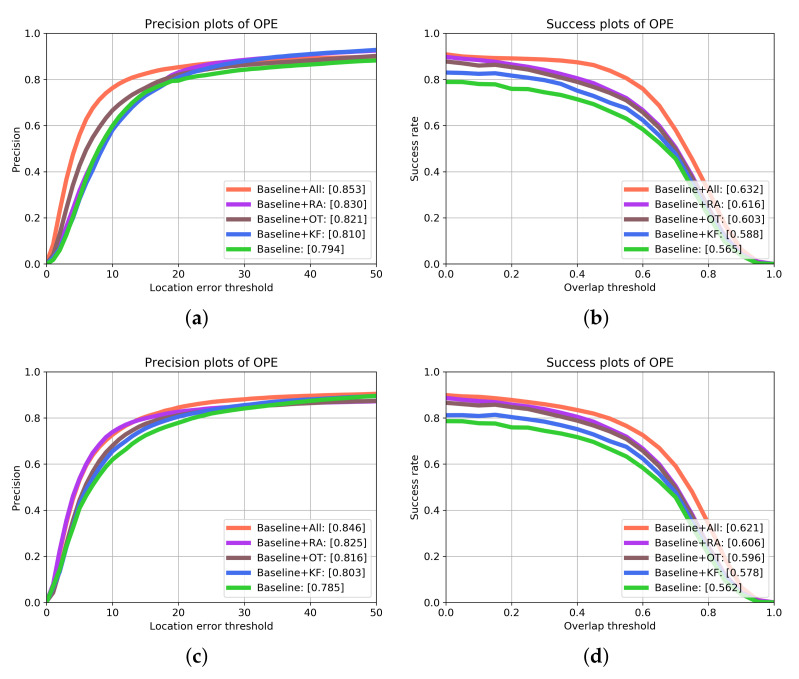
Overall results of ablation experiments by using one-pass-evaluation on the OTB-100 dataset. The upper and lower pairs are the results on OTB2013 and OTB2015 respectively.

The overall experimental results are shown in Figure 5. The left figure shows the experimental results of the accuracy measurement. The number in the legend is the tracking precision when the distance error threshold is 20. The right figure shows the total success rate of each tracker. The number in legend is the AUC (area under curve). In precision plots, Baseline+All obtains the optimal performance of 85.3%, 84.6% on OTB2013, OTB2015, respectively. Compared with the other four constructed trackers, the precision performance gains on OTB2013 are 2.3%, 3.2%, 4.3% and 5.9%, and those on OTB2015 are 2.1%, 3.0%, 4.3% and 6.1%, respectively. Similarly, Baseline+All obtains precision scores of 63.2%, 62.1% on OTB2013, OTB2015 in success plots. Compared with the other four trackers, the success gains on OTB2013 are 1.6%, 2.9%, 4.5% and 6.7%, and those on OTB2015 are 1.5%, 2.5%, 4.3% and 5.9%, respectively. We can see that KF has the least improvement in the performance of the benchmark tracker among the three modules. This is because the accuracy of the benchmark tracker is low, which makes the measurement error in the KF process larger and leads to suboptimal estimation results, which makes a single KF module improve the performance of the benchmark tracker less than the other three modules. The RA module contributes the most to the performance gain of the baseline tracker, this shows that reliable object information is very important for robust tracking process. OT module also plays an important role in improving the performance of the benchmark tracker. This is because the module always keeps a certain initial frame target information in the model. For occlusion, long sequence and other scenes, it can effectively keep the reliable information of the target and avoid tracking drift and failure.

The OTB dataset is manually tagged with 11 different attributes, which represents the challenging aspects. These attributes include—Illumination Variation, Occlusion, Fast Motion, Background Clutters, Out-of-Plane Rotation, Deformation, In-Plane Rotation, Low Resolution, Scale Variation, Motion Blur, Out-of-View. These subsets based on attributes play an important role in evaluating tracker performance and further improvement. Table 1 and Table 2 show the precision scores and AUC scores of each tracker on the 11 attribute based subset, respectively. We can see that Baseline+All has an absolute advantage over other trackers in all attribute subsets. Our proposed framework has improved the performance of the baseline tracker greatly, and the performance gain of the baseline tracker based on each module is consistent with the total result. This further confirms that reliable target information is the most important for the tracking process, and KF also provides important supplementary information for robust tracking.

### 4.3. Comparison with Other Trackers

In order to analyze and evaluate the proposed tracker more comprehensively, we compare it with other trackers on OTB and VOT datasets.

**OTB Dataset**. We compare our tracker with 18 latest methods: TLD [40], CSK [13], MOSSE [12], Struck [41], KCF [7], DSST [15], CFNet [11], Staple [42], SiamFC [24], SiamDCF [43], SiamTri [44], SRDCF [8], DLSSVM [45], CNN-SVM [46], ACFN [30], SRDCFad [47], DeepSRDCF [16], TRACA [48]. We also carried out experiments on OTB2013 and OTB2015, respectively.

**Figure 6:**
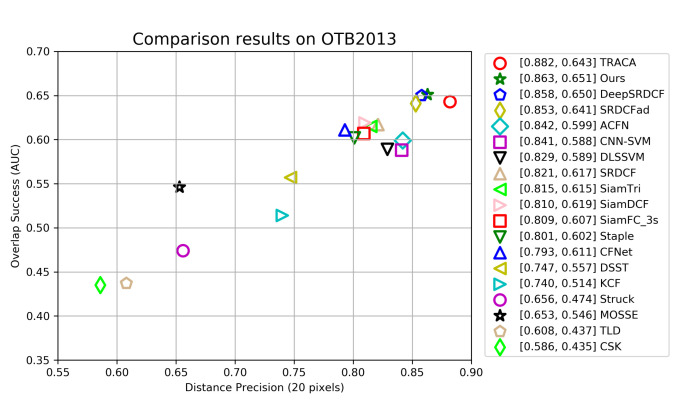
The precision and success scores of our tracker and other trackers on the OTB2013 dataset. The two columns of numbers in the legend represent the AUC score and the precision score at a threshold of 20 pixels. All trackers are sorted in the legend by precision scores.

**Figure 7:**
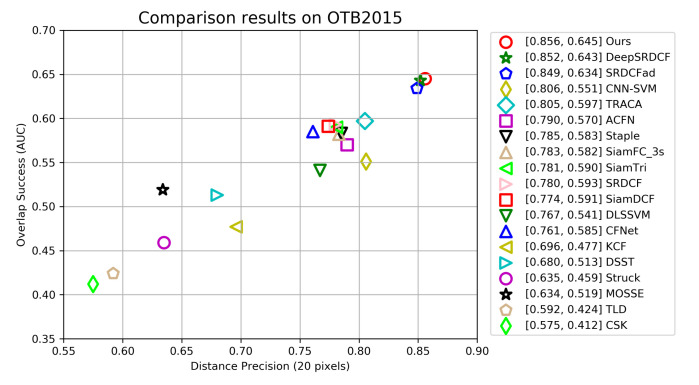
The precision and success scores of our tracker and other trackers on the OTB2015 dataset. The two columns of numbers in the legend represent the AUC score and the precision score at a threshold of 20 pixels. All trackers are sorted in the legend by precision scores.

Figure 6 shows the tracking performance of all trackers on benchmark OTB2013. Our tracker achieves the second-best performance in distance precision score of 86.3%, but the AUC score of 65.1% outperforms all 18 other trackers. The best tracker TRACA outperforms our tracker by 1.9% in distance precision, but its AUC performance is 0.8% lower than ours. Figure 7 illustrates the tracking performance of all trackers on OTB2015 dataset. We can see that our tracker’s AUC and DP scores are 85.6% and 64.5% respectively, which makes our tracker completely outperforms all other trackers in two indicators. The AUC and DP scores of the best performance tracker TRACA on OTB2013 decreased by 7.7% and 4.6% on OTB2015, respectively. The performance of ACFN on OTB2015 is also decreased by 5.2% and 2.9%. Different from many other trackers, the performance of our tracker on OTB2015 is slightly lower than that of OTB2013, which decreases by 0.7% and 0.6% respectively. This shows that our tracker can deal with complex tracking scene better and has high tracking robustness. Overall, the experiment results on two benchmarks demonstarte that our tracker performs well against other 18 tracker.

**Figure 8:**
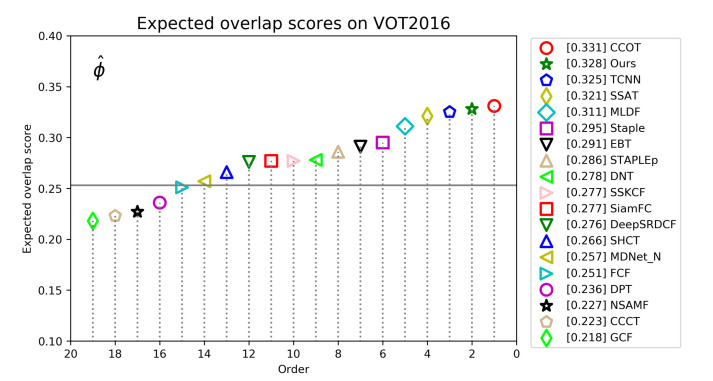
Expected average overlap scores ranking for compared trackers on the VOT2016 benchmark. The further to the right, the better the performance of the tracker.

**VOT2016 Dataset**. The VOT2016 dataset contains 60 short video sequences, and the accuracy (A), robustness (R) and expected average overlap (EAO) are three important criterion for evaluating trackers. In addition, EFO is often used to measure tracking speed. We compare our approach with 18 other state-of-the-art tracking algorithms on the VOT2016 benchmark. Figure 8 shows the EAO scores and rankings of all trackers on VOT2016. The best tracker is CCOT, with an EAO score of 0.331. Our tracker ranks second, with a performance slightly lower than that of CCOT, with an EAO score of 0.328. It is worth noting that the trackers above the horizontal line in the figure can be considered as state-of-the-art. Table 3 reports the detailed performance information about ours and several top trackers on VOT2016. Of all the trackers, our tracker ranked fourth in accuracy and first in robust. Although our tracker’s accuracy score is inferior to the top three, it is only 0.5% lower than the best tracker. Our tracker ranks first in robust, which shows that reliable target information and motion information are very important to the robustness of the tracker. Our tracker can adapt to a variety of challenging tracking scenarios.

**Figure 9:**
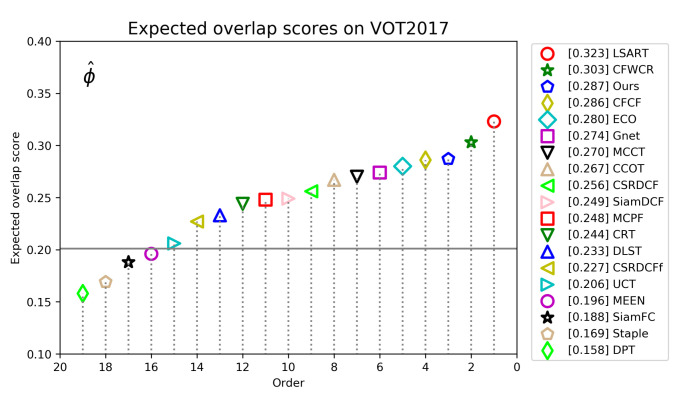
Expected average overlap scores ranking for compared trackers on the VOT2018 benchmark. The further to the right, the better the performance of the tracker.

**VOT2017 Dataset**. VOT2017 maintains 60 video sequences just like VOT2016. The difference is that VOT2017 removes 10 least challenging sequences from VOT2016, and adds 10 new sequences while keeping the overall attribute distribution unchanged. At the same time, it also re-calibrates the ground truth of all sequences. Figure 9 shows the EAO scores and rankings of all compared trackers on VOT2017. We can see that the best tracker is LSART with an EAO score of 0.323, while our tracker ranks third with an EAO score of 0.287. CCOT, the best tracker in VOT2016, has an EAO score of 0.267, which is 2.0% lower than our tracker. This is mainly due to the replacement of 10 new sequences, which makes the VOT2017 dataset more challenging than VOT2016, and our tracker has higher robustness in complex scenes, so our tracker performs better on VOT2017 than CCOT. Table 4 reports the detailed performance information about ours and 10 top trackers on VOT2017. We can see that our tracker ranked third in term of robustness with a score of 0.273, better than 0.318 of CCOT.

### 4.4. Quantitative Results

**Figure 10:**
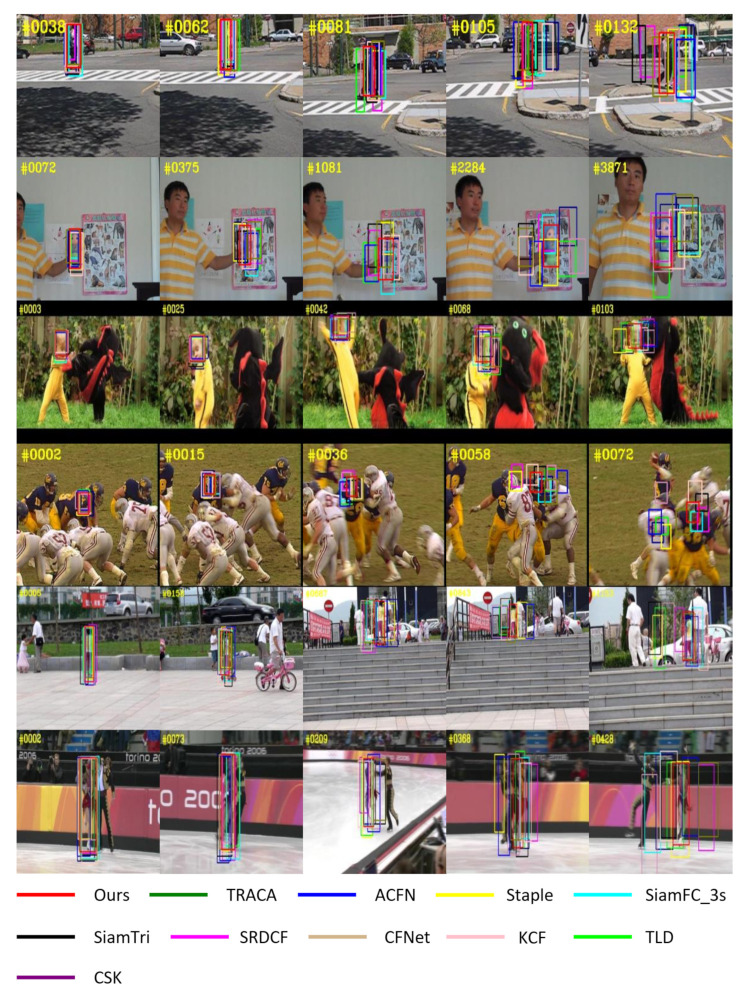
Quantitative tracking results of our tracker with other 10 trackers on OTB dataset. The video sequences are Couple, Doll, DragonBaby, Football1, Girl2 and Skating2_1 from top to bottom. The bottom illustration shows the colors for all trackers.

In order to analyze the tracking performance more intuitively, we compare our tracker with 10 other trackers on several challenging video sequences on OTB datasets, and give the quantitative tracking results in Figure 10. We can see that our tracker can accurately locate the target under the influence of occlusion, long sequence, distractors and other factors. It shows that our tracker can reliably keep the target information, and obtain the motion information between frames through KF module, which makes it possible to deal with a variety of complex scenes. So our tracker achieves the best performance in these challenging sequences.

## 5. Conclusions

In this paper, We propose a robust visual tracking framework which mainly includes three modules: reliability analysis module, reliable online training and update module, and KF module. The reliability analysis module is mainly used to analyze the tracking process and identify whether the training update step can be carried out to prevent the introduction of noise information. The reliable online training update module is mainly to fuse the information of the first frame and the current frame to maintain the most reliable target information in the tracking process. KF module models the motion information between frames, which provides important supplementary information for our tracker. The proposed method improves the tracking performance of the tracker in complex scenes such as appearance change, tracking drift and occlusion. We validate the proposed framework on several benchmark datasets. Our tracker achieves the second and first AUC scores on OTB2013 and OTB2015, respectively. On VOT2016 and VOT2017 datasets, our tracker is also at the top. The tracking results show that our tracker achieves state-of-the-art performance. However, we observed that our tracker cannot deal with the deformation of objects very well. In future work, we will continue to optimize our tracker.

## Figures and Tables

**Table 1:** Precision scores of ablation experiments on 11 attributes of OTB100.

	Baseline + All	Baseline + RA	Baseline + OT	Baseline + KF	Baseline
Illumination Variation	0.848	0.823	0.813	0.796	0.786
Occlusion	0.831	0.801	0.796	0.775	0.771
Fast Motion	0.763	0.742	0.741	0.726	0.706
Background Clutters	0.856	0.836	0.824	0.819	0.786
Out-of-Plane Rotation	0.828	0.811	0.795	0.791	0.782
Deformation	0.852	0.831	0.825	0.814	0.805
In-Plane Rotation	0.830	0.822	0.814	0.807	0.789
Low Resolution	0.798	0.776	0.765	0.758	0.737
Scale Variation	0.817	0.801	0.796	0.785	0.754
Motion Blur	0.788	0.765	0.753	0.750	0.736
Out-of-View	0.721	0.709	0.691	0.679	0.667

**Table 2:** Area under curve (AUC) scores of ablation experiments on 11 attributes of OTB100.

	Baseline + All	Baseline + RA	Baseline + OT	Baseline + KF	Baseline
lllumination Variation	0.603	0.582	0.579	0.564	0.556
Occlusion	0.598	0.581	0.572	0.552	0.539
Fast Motion	0.573	0.558	0.546	0.530	0.524
Background Clutters	0.628	0.612	0.609	0.597	0.583
Out-of-Plane Rotation	0.615	0.592	0.583	0.572	0.551
Deformation	0.606	0.586	0.574	0.559	0.531
In-Plane Rotation	0.624	0.608	0.582	0.583	0.568
Low Resolution	0.542	0.521	0.516	0.503	0.496
Scale Variation	0.581	0.571	0.559	0.542	0.532
Motion Blur	0.599	0.583	0.577	0.552	0.548
Out-of-View	0.532	0.519	0.503	0.498	0.482

**Table 3:** Detailed performance information about ours and several top tracker on VOT2016. Red, blue and green highlighted numbers indicate the 1st, 2nd and 3rd respectively.

	Ours	CCOT	TCNN	SSAT	MLDF	Staple	EBT	STAPLEp	DNT	SSKCF	SiamFC
EAO↑	0.328	0.331	0.325	0.321	0.311	0.295	0.291	0.286	0.278	0.277	0.277
Accuracy↑	0.552	0.539	0.554	0.577	0.490	0.544	0.465	0.557	0.515	0.547	0.549
Robust↓	0.230	0.238	0.268	0.291	0.233	0.378	0.252	0.368	0.329	0.373	0.382
EFO↑	10.16	0.507	1.049	0.475	1.483	11.14	3.011	44.77	1.127	29.15	5.444

**Table 4:** Detailed performance information about ours and several top tracker on vot2017. Red, blue and green highlighted numbers indicate the 1st, 2nd and 3rd respectively.

	Ours	LSART	CFWCR	CFCF	ECO	Gnet	MCCT	CCOT	CSRDCF	SiamDCF	MCPF
EAO↑	0.287	0.323	0.303	0.286	0.280	0.274	0.270	0.267	0.256	0.249	0.248
A↑	0.486	0.493	0.484	0.509	0.483	0.502	0.525	0.494	0.491	0.500	0.510
R↓	0.273	0.218	0.267	0.281	0.276	0.276	0.323	0.318	0.356	0.473	0.427

## Data Availability

Data of this research is available upon request via corresponding author.
